# Epidemiological Impact of Novel Preventive and Therapeutic HSV-2 Vaccination in the United States: Mathematical Modeling Analyses

**DOI:** 10.3390/vaccines8030366

**Published:** 2020-07-08

**Authors:** Houssein H. Ayoub, Hiam Chemaitelly, Laith J. Abu-Raddad

**Affiliations:** 1 Department of Mathematics, Statistics, and Physics, Qatar University, Doha 2713, Qatar; hayoub@qu.edu.qa; 2 Infectious Diseases Epidemiology Group, Weill Cornell Medicine–Qatar, Cornell University, Qatar Foundation–Education City, Doha 24144, Qatar; hsc2001@qatar-med.cornell.edu; 3 World Health Organization Collaborating Centre for Disease Epidemiology Analytics on HIV/AIDS, Sexually Transmitted Infections, and Viral Hepatitis, Weill Cornell Medicine–Qatar, Cornell University, Qatar Foundation–Education City, Doha 24144, Qatar; 4 Department of Healthcare Policy and Research, Weill Cornell Medicine, Cornell University, New York City, NY 10065, USA

**Keywords:** herpes simplex virus, genital herpes, vaccine, prevalence, incidence, mathematical model

## Abstract

This study aims to inform herpes simplex virus type 2 (HSV-2) vaccine development, licensure, and implementation by delineating the population-level impact of vaccination. Mathematical models were constructed to describe the transmission dynamics in presence of prophylactic or therapeutic vaccines assuming 50% efficacy, with application to the United States. Catch-up prophylactic vaccination will reduce, by 2050, annual number of new infections by 58%, incidence rate by 60%, seroprevalence by 21%, and avert yearly as much as 350,000 infections. Number of vaccinations needed to avert one infection was only 50 by 2050, 34 by prioritizing those aged 15–19 years, 4 by prioritizing the highest sexual risk group, 43 by prioritizing women, and 47 by prioritizing men. Therapeutic vaccination of infected adults with symptomatic disease will reduce, by 2050, annual number of new infections by 12%, incidence rate by 13%, seroprevalence by 4%, and avert yearly as much as 76,000 infections. Number of vaccinations needed to avert one infection was eight by 2050, two by prioritizing those aged 15–19 years, three by prioritizing the highest sexual risk group, seven by prioritizing men, and ten by prioritizing women. HSV-2 vaccination offers an impactful and cost-effective intervention to prevent genital herpes medical and psychosexual disease burden.

## 1. Introduction

Herpes simplex virus type 2 (HSV-2) infection is lifelong and one of the most prevalent sexually transmitted infections (STIs) [1,2,3]. The World Health Organization (WHO) estimated the number of persons living with HSV-2 globally at 491 million in 2016, equivalent to 13.2% of the world’s population aged 15–49 years [3]. Though low and middle income countries (LMICs) are most affected [1,3], large HSV-2 epidemics have been also documented in high income countries, such as the United States (US), with prevalent infections estimated at 50 million at present, and incident/new infections at over 600,000 every year [4].

The chronic nature of HSV-2 infection, with frequent and mostly unrecognized reactivations [5,6], its high infectiousness [7,8], and low threshold for sustainable transmission in the population [1,9,10], distinguishes its epidemiology from that of other STIs [10]. HSV-2 is a leading cause of genital herpes [1,11,12,13,14,15,16], and of genital ulcer disease (GUD)—painful conditions with serious consequences on sexual and reproductive health [17]. The latter conditions have been further associated with a range of psychosexual adverse outcomes [17,18,19,20]. HSV-2 can be passed vertically from mother-to-child leading to neonatal herpes, a rare but serious disease with high mortality risk [17,21]. Though with caveats [22], evidence suggests an epidemiologic synergy between HSV-2 and HIV infections [10,23,24,25], and a major role for HSV-2 in fueling the HIV epidemics especially in sub-Saharan Africa [10,26,27]. 

Controlling HSV-2 infection is integral to global efforts aimed at improving sexual and reproductive health [28,29]. Available prevention modalities, such as condoms and antiviral therapy, are insufficient to control infection spread, and therefore no specific national programs were set for HSV-2 prevention and control, including in high income countries such as the US [30,31,32]. However, the expensive direct medical costs of HSV-2 disease burden, estimated at $541 million per year in the US alone [33], highlight the critical need for HSV-2 vaccination as a strategic approach to control transmission and to curb the clinical, psychosexual, and economic disease burden of this infection [34].

Prophylactic and therapeutic HSV-2 vaccine candidates are currently in phase I and/or II trials [29,35,36]. In addition, a therapeutic vaccine candidate has already demonstrated sustained reductions in shedding and lesion rates over a 12-months duration, with no serious adverse events [37,38,39]. Given this progress, the WHO and global partners are spearheading development of a comprehensive business case for these vaccines [28,29,36] to catalyze stakeholders’ engagement and investment in vaccine development [28,29]. In this context, the WHO has recently called for mathematical modeling contributions to support the business case articulation of global health needs, vaccine preferred product characteristics (PPCs), vaccine potential impact, pathways and costs for vaccine development and administration, and expected cost-effectiveness and return on investment [28,29,36,40]. This business case is part of a global roadmap formulated to advance STI vaccine development and decision-making [41].

Building on a recently developed mathematical model characterizing the past, present, and future levels and trends of HSV-2 epidemics [4], and using nationally representative HSV-2 antibody prevalence (seroprevalence) data for the US over four decades [30,42,43,44,45,46], this study aims to assess the impact of HSV-2 vaccination in the US population, as an illustrative example of the public health benefits of a national vaccine program. The overarching goal of this study is to provide the scientific evidence necessary to rationalize a strategic approach that informs and accelerates the development of both prophylactic and therapeutic HSV-2 vaccines, at a critical time of such vaccines development [28]. Specifically, we assessed the impact of both a partially efficacious prophylactic vaccine, that reduces susceptibility to infection upon vaccination, and a partially efficacious therapeutic post-exposure vaccine that “treats” HSV-2 infection by reducing HSV-2 shedding frequency, thereby reducing HSV-2 symptomatic disease (genital lesions and recurrences).

## 2. Materials and Methods

### 2.1. Mathematical Model

**Figure 1:**
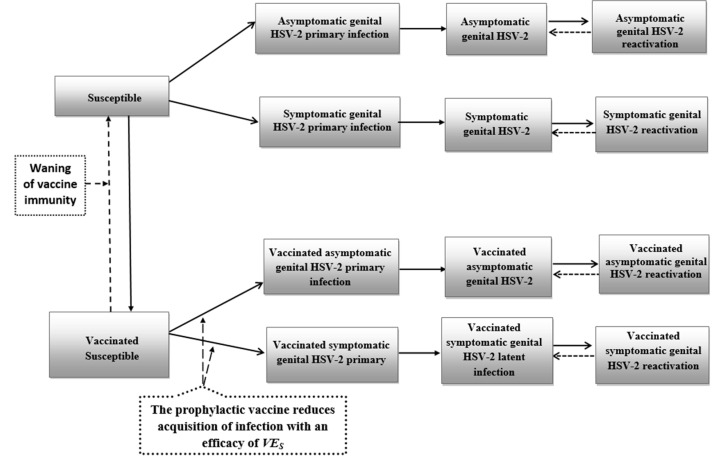
Conceptual diagram illustrating the effect of a prophylactic vaccine on HSV-2 acquisition. VE_S_ is defined as the proportional reduction in the susceptibility to infection among those vaccinated relative to those unvaccinated. Asymptomatic genital HSV-2 infection was defined as a person who is HSV-2 seropositive but never develops significant symptoms to warrant medical intervention, whereas symptomatic genital HSV-2 infection refers to an HSV-2 seropositive person who develops symptoms that may warrant medical intervention. In this figure, solid lines denote progression or forward movement from one population compartment to the next, while dashed lines denote backward movement from the present population compartment to the previous population compartment.

**Figure 2:**
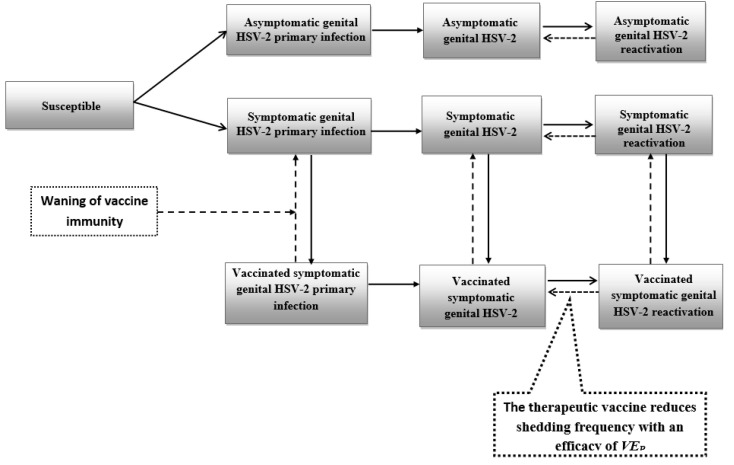
Conceptual diagram illustrating the effect of a therapeutic vaccine on reducing HSV-2 shedding frequency. VE_P_ is defined as the proportional reduction in shedding frequency among those vaccinated relative to those unvaccinated. Asymptomatic genital HSV-2 infection was defined as a person who is HSV-2 seropositive but never develops significant symptoms to warrant consideration of therapeutic vaccination/medical intervention, whereas symptomatic genital HSV-2 infection refers to an HSV-2 seropositive person who develops symptoms that may warrant consideration of therapeutic vaccination/medical intervention. In this figure, solid lines denote progression or forward movement from one population compartment to the next, while dashed lines denote backward movement from the present population compartment to the previous population compartment.

Two deterministic compartmental mathematical models were constructed to describe HSV-2 sexual transmission in the US population in presence of prophylactic (Figure 1 and Supplementary Material (SM) Figure S1) or therapeutic (Figure 2 and SM Figure S2) vaccination. The models adapted and extended a recently developed model that characterized the HSV-2 epidemic in the US from 1950–2050 [4], as informed by existing modeling approaches for STI vaccines [47,48,49,50,51,52,53,54,55,56], and a detailed review of HSV-2 vaccine models [40]. Models were structured by sex, age, and sexual activity, for broad application, and consisted of sets of coupled nonlinear differential equations.

Strata included sex, 20 five-year age groups spanning individuals 0–100 years of age, and five sexual risk groups with a hierarchy from lower to higher risk based on number of sexual partners over the last 12 months [57]. The distribution of sexual risk behavior followed a power-law function, as informed by sexual partner data [58] and a range of network and modeling analyses [59,60,61,62]. Age dependence of sexual activity was determined by sexual partner data [57], with sexual debut assumed at age ≥15 years. Sexual mixing by age and risk group was described by mixing matrices that included assortative (i.e., partners choosing partners from within their age or risk group) and proportionate (i.e., no preferential bias in choosing partners based on age or risk group) components, as informed by earlier modeling work [63,64,65].

The population was further stratified based on HSV-2 infection status, stage of infection, and vaccination status. Infection progression was modeled in terms of three stages: primary infection, latent infection, and infection reactivation, and varied between population groups based on absence/presence of symptoms and vaccination status (Figure 1 and Figure 2, and SM Figures S1 and S2). HSV-2 shedding, defining infectiousness, occurred only during primary infection and reactivations, regardless of presence of symptomatic disease. Further details on model structure and vaccination components can be found in SM. The model was coded, fitted, and analyzed in MATLAB R2018b [66].

### 2.2. Model Parameterization and Fitting

Model parameterization was based on current data for HSV-2 natural history and epidemiology. The model was calibrated through fitting to sex- and age- stratified HSV-2 seroprevalence data in the US from ten biennial rounds of the nationally representative and population-based National Health and Nutrition Examination Surveys (NHANES) 1988–2016 [57]. All surveys followed a standardized methodology [57], and were analyzed per NHANES standardized “survey methods and analytic guidelines,” with application of sampling weights [67]. Fitting to input data was performed using a non-linear least-square fitting technique, incorporating the Nelder-Mead simplex algorithm [68], as informed by earlier modeling work [64,65,69,70].

Parameterization of sexual risk behavior (into the five sexual risk groups) and of age dependence of sexual risk behavior were informed by NHANES data for the reported number of sexual partners in the last 12 months [57]. No risk compensation was assumed with HSV-2 vaccination. US population demographics and their future projections were obtained from the United Nations’ World Population Prospects database [71]. Further details on model parameters, values, and supporting evidence are in SM Tables S1 and S2.

### 2.3. Product Characteristics of Candidate Vaccines

The impact of two types of imperfect vaccines were assessed. The first is a prophylactic vaccine that reduces susceptibility to infection acquisition. Vaccination would be administered to a fraction of susceptible (HSV-2 seronegative) individuals, with efficacy *VE_S_* (“degree-type” protection [72]) defined as the proportional reduction in the susceptibility to infection among those vaccinated relative to those unvaccinated. No additional “breakthrough” effects, that is effects modulating the natural history of the infection for those vaccinated but who still acquire the infection, were assumed for this vaccine.

The second is a therapeutic vaccine that reduces shedding frequency, thus reducing genital HSV-2 reactivation/episode duration. Vaccination would be administered to a fraction of infected (HSV-2 seropositive) individuals with symptomatic disease, the likely mode of administration for this vaccine [28,29,36]. Symptomatic genital HSV-2 infection was defined here as an HSV-2 seropositive person who develops symptoms that warrant consideration of therapeutic vaccination/medical intervention, whereas asymptomatic genital HSV-2 infection refers to an HSV-2 seropositive person who never develops significant symptoms to warrant therapeutic vaccination. It was assumed that 25% of those seropositive develop some form of clinical disease, based on existing evidence [6,43,73,74,75,76]. Here, vaccine efficacy *VE_p_* is defined as the proportional reduction in shedding frequency among those vaccinated relative to those unvaccinated. Since this vaccine reduces shedding frequency, it indirectly also reduces HSV-2 transmission as those vaccinated will have less shedding time to pass the infection. We assumed implicitly that reduction in shedding implies proportional reduction in infectiousness.

We further assessed the impact assuming different durations of vaccine protection, *D*, defined as the total duration of protection that vaccination will elicit, through initial vaccination combined possibly with a booster [29].

### 2.4. Measures of Vaccine Impact

Direct and indirect public health benefits of vaccination were assessed. The direct impact results from the effects of the vaccine efficacies *VE_S_* and *VE_p_*. The indirect impact results from the reduction in the onward transmission of the infection. The total impact of the vaccine, that is the sum of direct and indirect effects, was estimated by comparing prevalence, incidence, and incidence rate at a given time in presence of vaccination, with that in the no-vaccination counter-factual scenario. Impact was also estimated by quantifying *effectiveness*, that is number of vaccinations needed to avert one infection over a specific time-horizon. This metric is essentially *cost-effectiveness* but with no costs included, as they are not yet available.

Vaccination impact was assessed at *VE_S_* or *VE_p_* of 50%. This choice was motivated, for *VE_S_*, by current data on vaccine candidates [29,77], the indicated WHO PPCs [36], and the minimum efficacy level for an HSV-2 vaccine to be licensed and administrated [48]; and for *VE_p_* by current data on vaccine candidates [38,39] and the indicated WHO PPCs [36]. Duration of protection was assumed at 20 years in case of a prophylactic vaccine, and 10 years in case of a therapeutic vaccine.

### 2.5. Vaccination Program Scenarios

For the prophylactic vaccine, the main scenario was that of “catch-up vaccination,” defined as vaccine administration to all uninfected adults 15–49 years of age in 2020, with coverage scale-up at a constant rate to 80% by 2030, but without the additional vaccination of younger age groups. An alternative “single-sex vaccination” scenario was also investigated by restricting vaccination to women 15–49 years of age.

Additional scenarios included (1) vaccination of only adolescents 10–14 years of age, building on existing human papillomavirus (HPV) vaccination programs and available vaccine-delivery infrastructure [29,36], (2) vaccination of only infants with a vaccine that elicits protection for 30 years instead of 20 years, given the possibility of an efficacious vaccine against both HSV-1 and HSV-2 (for which infant vaccination is most relevant [29]).

For the therapeutic vaccine, the investigated scenario was vaccination of infected persons *with symptomatic disease* in 2020, with coverage scale-up at a constant rate to 80% by 2030.

Number of vaccinations needed to avert one infection (that is effectiveness) of prophylactic and therapeutic vaccines was investigated through different sub-population prioritization schemes based on sex, age, and sexual risk behavior.

### 2.6. Sensitivity Analyses

For each of the prophylactic and therapeutic vaccines, effectiveness was assessed at broad values for *VE_S_*, *VE_p_*, and duration of vaccine protection. Impact of prophylactic vaccination was also assessed at different vaccine coverage levels.

### 2.7. Uncertainty Analysis

A multivariable uncertainty analysis was conducted to determine the range of uncertainty around model-predicted vaccine effectiveness with respect to variations in the bio-behavioral parameters of the models (SM Tables S1 and S2). For each vaccine type, 500 model runs were performed, where in each run, Latin hypercube sampling [78,79] is applied in the selection of parameter values from ranges that assume ±40% uncertainty around parameters’ point estimates, and the model refitted to input data. Means and associated 95% uncertainty intervals (UIs) for the vaccine effectiveness were calculated at each time point across these runs.

## 3. Results

### 3.1. Prophylactic Vaccine

**Figure 3:**
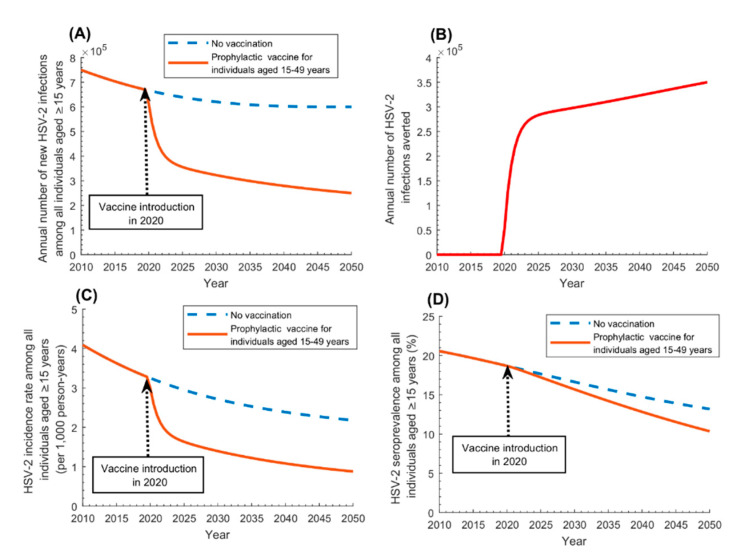
Impact of prophylactic HSV-2 vaccination administered to uninfected adults aged 15–49 years (catch-up vaccination) on HSV-2 infection measures in the population aged ≥15 years. Impact of the prophylactic vaccine on (**A**) annual number of new HSV-2 infections, (**B**) annual number of HSV-2 infections averted, (**C**) HSV-2 incidence rate, and (**D**) HSV-2 seroprevalence, among those aged ≥15 years. The prophylactic vaccine is introduced in 2020, with its coverage scaled up to 80% by 2030, and maintained at this level thereafter. Duration of vaccine-induced protection is 20 years and *VE_S_* is 50%.

**Figure 4:**
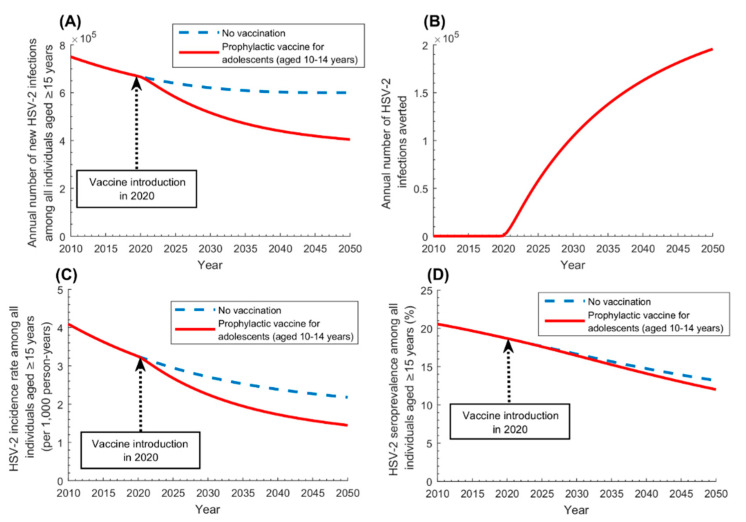
Impact of prophylactic HSV-2 vaccination administered to adolescents aged 10–14 years (adolescents’ vaccination) on HSV-2 infection measures in the population aged ≥15 years. Impact of the prophylactic vaccine on (**A**) annual number of new HSV-2 infections, (**B**) annual number of HSV-2 infections averted, (**C**) HSV-2 incidence rate, and (**D**) HSV-2 seroprevalence, among those aged ≥15 years. The prophylactic vaccine is introduced in 2020, with its coverage scaled up to 80% by 2030, and maintained at this level thereafter. Duration of vaccine-induced protection is 20 years and *VE_S_* is 50%.

Figure 3 and Figure 4 and SM Figures S3 and S4 show the impact of prophylactic vaccination assuming different scenarios. Catch-up vaccination (vaccinating susceptible adults 15–49 years of age; Figure 3) will yield, by 2050, a reduction of 58% in annual number of new infections, 60% in incidence rate, and 21% in seroprevalence. The annual number of infections averted was 297,700 in 2030, 323,300 in 2040, and 350,100 in 2050, and the cumulative number of infections averted (by 2050) was 9,167,400. SM Figure S7A shows the vaccine coverage scale-up over time.

Meanwhile, single-sex vaccination (vaccinating susceptible women 15–49 years of age; SM Figure S3) will yield, by 2050, a reduction of 31% in annual number of new infections, 32% in incidence rate, and 12% in seroprevalence. The annual number of infections averted was 167,600 in 2030, 173,700 in 2040, and 183,200 in 2050, and the cumulative number of infections averted (by 2050) was 5,048,200.

Similarly, adolescents’ vaccination (vaccinating individuals 10–14 years of age; Figure 4) will yield, by 2050, a reduction of 33% in annual number of new infections, 34% in incidence rate, and 9% in seroprevalence. The annual number of infections averted was 104,400 in 2030, 162,700 in 2040, and 195,700 in 2050, and the cumulative number of infections averted (by 2050) was 3,773,200.

Infants’ vaccination (vaccinating newborns; SM Figure S4) will yield, by 2050, a reduction of 21% in annual number of new infections, 21% in incidence rate, and 3% in seroprevalence. The annual number of infections averted was 53,400 in 2040 and 124,200 in 2050, and the cumulative number of infections averted (by 2050) was 1,092,800.

**Figure 5:**
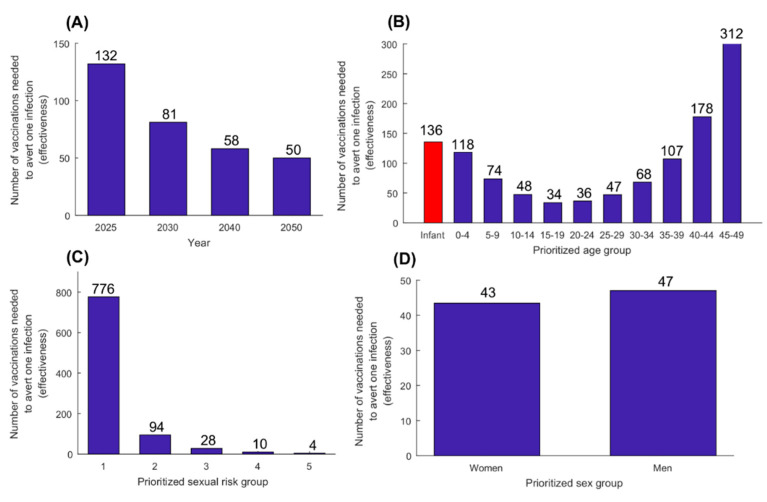
Effectiveness of prophylactic vaccination. Number of vaccinations needed to avert one infection (**A**) at different time points, and in 2050 (**B**) by prioritizing different age groups for vaccination, (**C**) by prioritizing different sexual risk groups, and (**D**) by prioritizing women as opposed to men. The prophylactic vaccine is introduced in 2020, with its coverage scaled up to 80% by 2030, and maintained at this level thereafter. Duration of vaccine-induced protection is 20 years and *VE_S_* is 50%.

Figure 5 shows results for the prophylactic vaccine effectiveness assessed using the catch-up vaccination scenario. Number of vaccinations needed to avert one infection was 132 by 2025 and 50 by 2050. Prioritizing those 15–19 years of age was most effective with only 34 vaccinations needed to avert one infection by 2050, while prioritizing those 45–49 years of age was least effective with 312 vaccinations needed to avert one infection. Vaccinating infants was not effective with 136 vaccinations needed to avert one infection.

Higher effectiveness was reached by prioritizing individuals with higher sexual risk behavior—only four vaccinations were needed to avert one infection by 2050 by prioritizing the highest sexual risk group (say female sex workers or men who have sex with men). Prioritizing women was slightly more effective than prioritizing men with 43 and 47 vaccinations needed to avert one infection by 2050, respectively. Single-sex vaccination still benefited both women and men. Single-sex vaccination of women yielded 40% incidence reduction in women and 16% in men, whereas single-sex vaccination of men yielded 44% incidence reduction in men and 28% in women.

SM Figures S5A and S6A illustrate results of sensitivity analyses assessing the impact of varying *VE_S_* and the vaccine duration of protection, respectively, on vaccine effectiveness. Number of vaccinations needed to avert one infection decreased steadily as each of *VE_S_* or the duration of protection increased. SM Figure S8 illustrates results of the sensitivity analysis for the impact of prophylactic vaccine coverage on annual incidence reduction. The impact of the vaccine was found to increase linearly with increasing vaccine coverage.

SM Figure S9A shows results of the uncertainty analysis assessing the robustness of model predictions for the prophylactic vaccine effectiveness. Results affirmed findings of favorable effectiveness for this vaccine.

### 3.2. Therapeutic Vaccine

**Figure 6:**
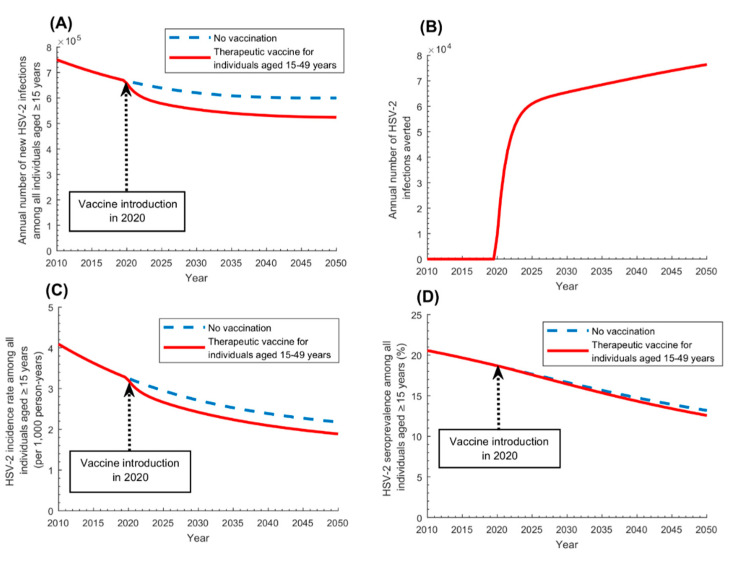
Impact of therapeutic HSV-2 vaccination administered to infected persons with symptomatic disease aged 15–49 years on HSV-2 infection measures in the population aged ≥15 years. Impact of the therapeutic vaccine on (**A**) annual number of new HSV-2 infections, (**B**) annual number of HSV-2 infections averted, (**C**) HSV-2 incidence rate, and (**D**) HSV-2 seroprevalence, among those aged ≥15 years. The therapeutic vaccine is introduced in 2020, with its coverage scaled up to 80% by 2030, and maintained at this level thereafter. Duration of vaccine-induced protection is 10 years and *VE_P_* is 50%.

Figure 6 shows the impact of therapeutic vaccination that is vaccinating infected adults 15–49 years of age with symptomatic disease. This scenario will yield, by 2050, a reduction of 12% in annual number of new infections, 13% in incidence rate, and 4% in seroprevalence. The annual number of infections averted was 65,500 in 2030, 71,300 in 2040, and 76,400 in 2050, and the cumulative number of infections averted (by 2050) was 1,998,200. SM Figure S7B shows the vaccine coverage scale-up over time.

**Figure 7:**
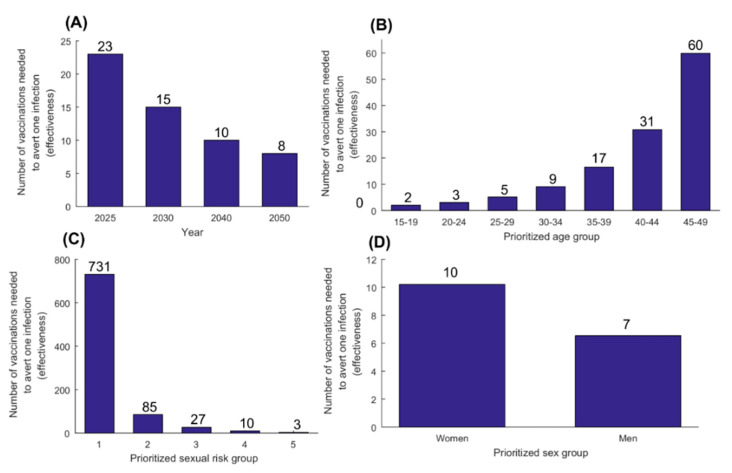
Effectiveness of therapeutic vaccination. Number of vaccinations needed to avert one infection (**A**) at different time points, and in 2050 (**B**) by prioritizing different age groups for vaccination, (**C**) by prioritizing different sexual risk groups, and (**D**) by prioritizing women as opposed to men. The therapeutic vaccine is introduced in 2020, with its coverage scaled up to 80% by 2030, and maintained at this level thereafter. Duration of vaccine-induced protection is 10 years and *VE_P_* is 50%.

Figure 7 shows results of the therapeutic vaccine effectiveness. Number of vaccinations needed to avert one infection was 23 by 2025 and 8 by 2050. Prioritizing those 15–19 years of age was most effective with only two vaccinations needed to avert one infection by 2050, while prioritizing those 45–49 years of age was least effective with 60 vaccinations needed to avert one infection.

Prioritizing individuals with higher sexual risk behavior yielded higher effectiveness—only three vaccinations were needed to avert one infection by 2050 by prioritizing the highest sexual risk group. Prioritizing men was more effective than prioritizing women with 7 and 10 vaccinations needed to avert one infection by 2050, respectively—a pattern that is opposite to that of the prophylactic vaccine (Figure 5D). Yet, single-sex vaccination still benefited both men and women. Single-sex vaccination of men yielded 2.7% incidence reduction in men but 7.9% reduction in women, whereas single-sex vaccination of women yielded 5.7% incidence reduction in women but 9.3% reduction in men. The larger impact for the *unvaccinated* sex is a consequence of the therapeutic vaccine reducing transmission, and not acquisition, of the infection.

SM Figures S5B and S6B illustrate results of sensitivity analyses assessing the impact of varying *VE_p_* and the vaccine duration of protection, respectively, on vaccine effectiveness. Number of vaccinations needed to avert one infection decreased steadily as each of *VE_p_* or the duration of protection increased.

SM Figure S9B shows results of the uncertainty analysis assessing the robustness of model predictions for the therapeutic vaccine effectiveness. Results affirmed findings of high effectiveness for this vaccine.

## 4. Discussion

This study provided an in-depth quantitative assessment for the impact and effectiveness of both prophylactic and therapeutic vaccination for HSV-2 infection. Findings demonstrated substantial gains in curbing HSV-2 infection and disease burden with either of these vaccines, and with relatively small number of vaccinations needed to avert one infection, therefore suggestive of high cost-effectiveness for these vaccines.

The results showed that even a partially efficacious prophylactic vaccine, with *VE_S_* of only 50%, would achieve 60% reduction in annual number of infections by 2050, that is averting as much as 350,000 infections every year (Figure 3). The vaccine was also cost-effective, with about 50 vaccinations needed to avert one infection (Figure 5A). These findings highlight the value of prophylactic vaccination for an infection for which there is still no specific national program for its prevention and control [30,31,32].

Prophylactic *catch-up* vaccination was the strategy yielding the most immediate impact, whether extended to the entire 15–49 years-old population (Figure 3), or to a single sex, say women (SM Figure S3). Best effectiveness, however, was attained by vaccinating those 15–19 years of age (Figure 5B), as they are vaccinated at entry of the age of highest incidence rate and are protected by the vaccine throughout their sexual lifetime. Higher effectiveness was also attained by vaccinating those at highest risk of infection, such as female sex workers and men who have sex with men (Figure 5C). Although HSV-2 infection is prevalent among the general population, unlike other STIs such as gonorrhea or syphilis, which are mostly concentrated in populations at high risk [10], prioritizing high risk populations for HSV-2 vaccination still yielded substantial gains in effectiveness. Effectiveness was also higher by prioritizing women, given their higher seroprevalence compared to men, but the differential in effectiveness was not substantial (Figure 5D). Regardless of whom will be prioritized for vaccination, the other sex will be also indirectly protected, as the vaccine will interrupt HSV-2 chains of transmission in the population. Adolescent and infant vaccinations were impactful but required longer time horizons for the impact to materialize (Figure 4 and SM Figure S4). Still, other factors, such as the availability of childhood/adolescent immunization programs and the feasibility of vaccine delivery, may warrant consideration of infant/adolescent vaccination.

The most striking finding of this study was the impact of the therapeutic vaccine, highlighting its utility as an intervention (Figure 6). This vaccine will not be indicated for its public health effects, but for its clinical benefits to the affected individual, as it would alleviate symptoms of genital herpes therefore improving the quality of life. The vaccine, however, can still have a considerable impact on infection transmission at the population level, despite the smaller scale of the public health benefits compared to the prophylactic vaccine, and the limited reach to only those with symptomatic disease. Indeed, by 2050, a therapeutic vaccine with an efficacy of only 50% would avert 12% of the annual number of infections (Figure 6A), that is averting as much as 76,000 infections every year (Figure 6B).

Importantly, therapeutic vaccination was very effective with only eight vaccinations needed to avert one infection (Figure 7A). Effectiveness can be further optimized by prioritizing those 15–19 years of age, with only two vaccinations needed to avert one infection (Figure 7B), or by prioritizing those at highest risk of infection, such as female sex workers and men who have sex with men, with only three vaccinations needed to avert one infection (Figure 7C). Opposite to prophylactic vaccination, therapeutic vaccination of men was more effective than vaccination of women, with 30% less vaccinations needed to avert one infection (Figure 7D). This differential effect by sex is explained by the therapeutic vaccine reducing the risk of infection transmission rather than infection acquisition. Indeed, with the vaccine *directly* blocking the transmission from men to women, who are more susceptible to infection acquisition, more infections are averted in the population for less vaccinations.

Findings demonstrated the role of indirect effects of vaccination in limiting the onward transmission of the infection and reducing HSV-2 infection burden in the population at large. For instance, women-only prophylactic vaccination will reduce HSV-2 incidence among men by 16% by 2050, even though men will not benefit directly from the vaccine. These indirect effects aggregate over time, and increase in importance the longer is the duration of the vaccination program. Of note that, for both vaccination types, the scale of public health benefits increases with vaccine efficacy (SM Figure S5), whether *VE_S_* or *VE_p_*, and importantly with the duration of protection elicited by the vaccine (SM Figure S6). Vaccine deployment strategy is another critical factor, as the magnitude of the impact varies based on which age group, risk group, or sex to be prioritized for vaccination (Figure 5; Figure 7).

While this study demonstrated the utility of both prophylactic and therapeutic HSV-2 vaccines, it may have underestimated the public health benefits, since only the epidemiological impact on HSV-2 infection was assessed, without factoring consequential benefits on other disease outcomes such as neonatal herpes [21], or other infections such as HIV or HSV-1. A potential important gain from HSV-2 vaccination, that is of particular relevance to LMICs, is perhaps the prevention of HIV infection [27,35,55]. The latter, however, should be subject to further investigation as although the vaccine may reduce the risk of HIV acquisition by reducing genital inflammation or shedding [35,80], the reverse effect is (theoretically) still possible with the influx of T-cells to the genital tract possibly increasing the risk of HIV acquisition [35,80,81]. Meanwhile, with HSV-1 and HSV-2 viruses sharing >83% of their genome and >85% of their protein profile [82], and considering evidence suggesting an epidemiologic interaction between the two infections [83,84], it is possible that an HSV-2 vaccine could have protective effects against HSV-1 infection. Indeed, the Herpvac vaccine, initially designed to prevent HSV-2 infection, had no effect on HSV-2 infection but reduced the incidence of genital HSV-1 disease by 58% and HSV-1 infection by 32% [85].

A prophylactic HSV-2 vaccine may also have “therapeutic” or “breakthrough” effects, in the form of additional efficacies against HSV-2 infection that were not assumed in our study. Motivated by data on animal models [86,87], these effects are especially important when *VE_S_* is low, making more room for these efficacies to leave an impact. For instance, the vaccine may have an efficacy against infectiousness (*VE_I_*; defined as the proportional reduction in infectiousness among those vaccinated, but still acquire the infection, relative to those unvaccinated) [47,48,49]. This is supported by the existence of a threshold for genital viral load to lead to transmission in a sexual act [88], and therefore a possible additional vaccine effect in reducing genital viral load during a shedding episode [49]. A prophylactic vaccine may also reduce HSV-2 shedding for those vaccinated but still acquire the infection, a similar mechanism of action to that of the therapeutic vaccine (*VE_p_*) [49,86,87]. Lastly, the assumed efficacy in the present study of only 50% may underestimate that for the eventually developed vaccine, especially so for the case of the therapeutic vaccine considering recent developments [89]. It is worth noting here that the minimum efficacy needed for vaccine licensing could be lower for therapeutic vaccines than for prophylactic vaccines, given the direct and immediate benefits of therapeutic vaccination on quality of life.

Our findings demonstrate the need for more and accelerated investment in HSV-2 vaccine development, more so that there is increasing optimism that developing a vaccine is feasible in the near future. Despite setbacks over three decades [77,85,90,91,92], optimism is grounded on progress in basic science and results of vaccine candidates currently in phase I and/or II trials [29,35,93]. Optimism is also grounded on several lines of evidence suggesting vaccine feasibility [35], including improved understanding of HSV immunology [35,40,94,95], growing knowledge of the optimal combination of antigens and adjuvants that could lead to vaccine protection [77,85,93,95,96,97,98,99,100,101], success and availability of both prophylactic and therapeutic vaccines against varicella zoster virus (VZV) [35,102,103], which is a closely related alpha-herpes virus, success and availability of animal herpes vaccines such as the bovine herpesvirus-1 [104] and the suid herpesvirus-1 (pseudorabies virus) [35,105], demonstration that intramuscular vaccination can induce genital mucosal immunity [35], as is the case for HPV vaccination [106], and the partial protection in the Herpvac trial against HSV-1 infection and genital disease [35,85] given the strong homology between HSV-1 and HSV-2 viruses [17,82].

Our study has limitations. The vaccine mechanism of action was assumed independent of HSV-1 infection status, but evidence suggests possible interactions between the two infections that may complicate transmission dynamics and assessment of vaccine impact [40,83,84]. An example is the lower risk of symptomatic disease among those HSV-1 seropositive who acquire HSV-2 infection [74]. The study did not assess vaccination impact on other disease outcomes or infections, such as neonatal herpes, HIV incidence, or HSV-1 incidence. The vaccine was also explored as a standalone intervention, regardless of the presence of other interventions. The study further assumed that the therapeutic vaccine efficacy persists for 10 years, however, the vaccine that may eventually be developed may not persistently eliminate viral shedding for a long duration. The study also did not explore the impact of therapeutic vaccination for the large asymptomatic HSV-2 infected population. Vaccine impact was assessed in a specific national setting, potentially limiting generalizability of the results to other settings at higher seroprevalence, such as in sub-Saharan Africa [1], or lower seroprevalence, such as in the Middle East and North Africa [3,107,108]. While this study provides estimates for the vaccine impact, it does not address complexities in vaccine implementation. For instance, a therapeutic vaccine may need to be implemented within the healthcare system and may require development of a sensitive and specific diagnostic test that can be feasibly implemented in LMICs [29].

Our study has strengths. We used an elaborate dynamical mathematical model to capture different heterogeneities and intricacies in the non-linear transmission dynamics, thus factoring the sexual contact structure and the biology of the infection in terms of natural history, susceptibility, and transmissibility, as well as accounting for the population-level benefits of the vaccine beyond the direct benefits to vaccinated individuals [28,72,109]. The model was stratified by sex, age, and sexual activity, that not only allowed a realistic description of the epidemiology, but also facilitated investigation of the vaccine impact by sub-population prioritization. The model was robustly calibrated for a specific national epidemic [4], thereby generating estimates and projections that are representative of the demographic diversity in the population at large. The model was anchored on high quality data for HSV-2 natural history and transmission, and the impact was assessed for different vaccine characteristics and vaccination strategies, to allow a detailed and nuanced understanding of the epidemiological impact. Of note that the presented herpes vaccination models addressed key limitations that have been recently highlighted in earlier models [40]. Uncertainty and sensitivity analyses were further conducted for a rigorous and broad assessment of vaccination impact with analyses confirming model predictions (SM Figures S5, S6, S8 and S9).

## 5. Conclusions

In conclusion, a quantitative investigation of the impact of HSV-2 vaccination in the US was conducted, and novel insights were delineated. A striking finding is that a therapeutic vaccine can have significant population-level benefits, even though it is indicated for only its individual-level clinical benefits. Such vaccine with intermediate efficacy can reduce HSV-2 incidence by >10%, averting 76,000 infections per year, and at very high effectiveness with only eight vaccinations needed to avert one infection. Meanwhile, a prophylactic vaccine of intermediate efficacy can reduce HSV-2 incidence by >50%, averting >350,000 infections per year, and at high effectiveness with only 50 vaccinations needed to avert one infection. The impact of these vaccines can also be optimized by prioritizing young adults and those at higher risk of infection. Mass catch-up vaccination, or alternatively single-sex catch-up vaccination, will be essential to achieve a sizable impact in the short-run, as adolescent or infant vaccination will require long-term implementation before effects materialize. These findings demonstrate the criticality of rapid development of prophylactic and therapeutic vaccines to control transmission and to prevent genital herpes medical and psychosexual disease burden.

## Supplementary Materials

The following are available online at https://www.mdpi.com/2076-393X/8/3/366/s1, Figure S1: Schematic diagram describing the structure of the HSV-2 mathematical model incorporating a prophylactic vaccine. Figure S2. Schematic diagram describing the structure of the HSV-2 mathematical model incorporating a therapeutic vaccine. Figure S3. Impact of prophylactic HSV-2 vaccination administered to susceptible women aged 15–49 years (single-sex vaccination) on HSV-2 infection measures in the population aged ≥15 years. Figure S4. Impact of prophylactic HSV-2 vaccination administered to infants (infants’ vaccination) on HSV-2 infection measures in the population aged ≥15 years. Figure S5. Sensitivity analysis assessing the effectiveness of prophylactic and therapeutic vaccination to a range of vaccine efficacies. Figure S6. Sensitivity analysis assessing the effectiveness of prophylactic and therapeutic vaccination to a range of vaccine-induced protection durations. Figure S7. Vaccine coverage of (A) the catch-up prophylactic vaccination scenario, and (B) the therapeutic vaccination scenario. Figure S8. Impact of prophylactic HSV-2 vaccination administered to uninfected adults aged 15–49 years (catch-up vaccination) on the reduction in the annual number of new HSV-2 infections assuming different levels of vaccine coverage. Figure S9. Uncertainty analysis. Table S1. Definitions of symbols in the equations of the mathematical models. Table S2. Model assumptions in terms of parameter values.
