# Supplementary material for: Epidemiological Impact of Novel Preventive and Therapeutic HSV-2 Vaccination in the United States: Mathematical Modeling Analyses
Source: Vaccines (Basel). 2020 Jul 8;8(3):366. doi: 10.3390/vaccines8030366 (PMC7564812; doi:10.3390/vaccines8030366)
Supplement: Supplementary file 1 [file vaccines-08-00366-s001.pdf]

Supplementary Material

Epidemiological Impact of Novel Preventive and Therapeutic HSV-2 Vaccination in the United States: Mathematical Modeling Analyses

Houssein H. Ayoub[1], Hiam Chemaitelly[2,3], and Laith J. Abu-Raddad[2,3,4*]

[1]Department of Mathematics, Statistics, and Physics, Qatar University, Doha 2713, Qatar; hayoub@qu.edu.qa (H.H.A.)

[2]Infectious Diseases Epidemiology Group, Weill Cornell Medicine–Qatar, Cornell University, Qatar Foundation – Education City, Doha 24144, Qatar; hsc2001@qatar-med.cornell.edu (H.C.); lja2002@qatar-med.cornell.edu (L.J.A.)

[3]World Health Organization Collaborating Centre for Disease Epidemiology Analytics on HIV/AIDS, Sexually Transmitted Infections, and Viral Hepatitis, Weill Cornell Medicine–Qatar, Cornell University, Qatar Foundation – Education City, Doha 24144, Qatar; hsc2001@qatar-med.cornell.edu (H.C.); lja2002@qatar-med.cornell.edu (L.J.A.)

[4]Department of Healthcare Policy and Research, Weill Cornell Medicine, Cornell University, New York City, NY 10065, USA; lja2002@qatar-med.cornell.edu (L.J.A.)

Reprints or correspondence:

Professor Laith J. Abu-Raddad, Infectious Disease Epidemiology Group, Weill Cornell Medicine - Qatar, Qatar Foundation - Education City, P.O. Box 24144, Doha, Qatar. Telephone: +(974) 4492-8321. Fax: +(974) 4492-8333. E-mail: lja2002@qatar-med.cornell.edu.

1. Mathematical model

Model equations

We developed two deterministic compartmental mathematical models, based on adaptation and extension of the herpes simplex virus type 2 (HSV-2) transmission model in Reference [1], to describe HSV-2 transmission dynamics in a population in presence of either a prophylactic vaccine (Figure S1) or a therapeutic vaccine (Figure S2). The models were expressed in terms of systems of coupled nonlinear differential equations that stratify the population into compartments according to sex (males, females), age (20 5-year age classifications), sexual risk group (5 sexual risk groups), HSV-2 status (uninfected, infected asymptomatic, infected symptomatic), and stage of infection (primary, latent, infection reactivation), and vaccination status (unvaccinated, vaccinated).

Figure S1. Schematic diagram describing the structure of the HSV-2 mathematical model incorporating a prophylactic vaccine.

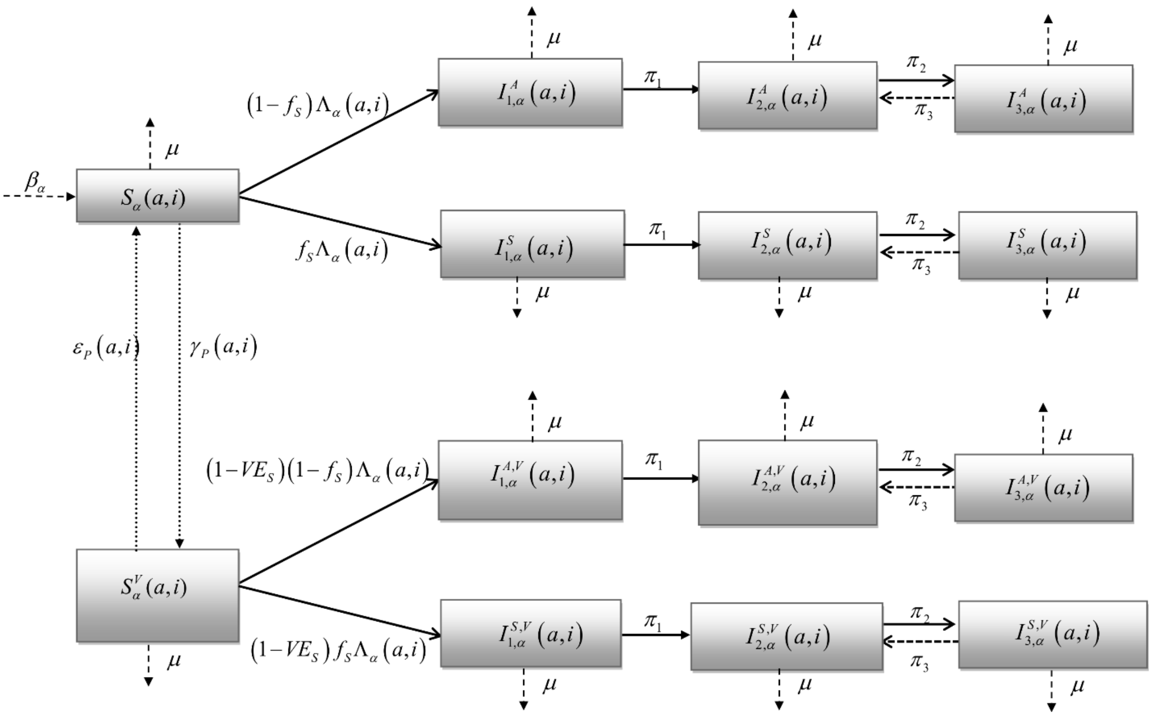

The prophylactic HSV-2 vaccine model was expressed using the following equations:

Unvaccinated population:

$$\frac{dS_\alpha(a,i)}{dt}=\beta_\alpha(t,a)N_\alpha(i)+\eta(a-1)S_\alpha(a-1,i)+\varepsilon_P(a,i)S_\alpha^V(a,i)-\left(\eta(a)+\mu_\alpha(a)+\Lambda_\alpha(a,i)+\gamma_P(a,i)\right)S_\alpha(a,i)$$

$$\frac{dI_{1,\alpha}^A(a,i)}{dt}=\left(1-f_S\right)\Lambda_\alpha(a,i)S_\alpha(a,i)+\eta(a-1)I_{1,\alpha}^A(a-1,i)-\left(\eta(a)+\mu_\alpha(a)+\pi_1\right)I_{1,\alpha}^A(a,i)$$

$$\frac{dI_{2,\alpha}^A(a,i)}{dt}=\pi_1 I_{1,\alpha}^A(a,i)+\eta(a-1)I_{2,\alpha}^A(a-1,i)+\pi_3 I_{3,\alpha}^A(a,i)-\left(\eta(a)+\mu_\alpha(a)+\pi_2\right)I_{2,\alpha}^A(a,i)$$

$$\frac{dI_{3,\alpha}^A(a,i)}{dt}=\pi_2 I_{2,\alpha}^A(a,i)+\eta(a-1)I_{3,\alpha}^A(a-1,i)-\left(\eta(a)+\mu_\alpha(a)+\pi_3\right)I_{3,\alpha}^A(a,i)$$

$$\frac{dI_{1,\alpha}^S(a,i)}{dt}=f_S\Lambda_\alpha(a,i)S_\alpha(a,i)+\eta(a-1)I_{1,\alpha}^S(a-1,i)-\left(\eta(a)+\mu_\alpha(a)+\pi_1\right)I_{1,\alpha}^S(a,i)$$

$$\frac{dI_{2,\alpha}^S(a,i)}{dt}=\pi_1 I_{1,\alpha}^S(a,i)+\eta(a-1)I_{2,\alpha}^S(a-1,i)+\pi_3 I_{3,\alpha}^S(a,i)-\left(\eta(a)+\mu_\alpha(a)+\pi_2\right)I_{2,\alpha}^S(a,i)$$

$$\frac{dI_{3,\alpha}^S(a,i)}{dt}=\pi_2 I_{2,\alpha}^S(a,i)+\eta(a-1)I_{3,\alpha}^S(a-1,i)-\left(\eta(a)+\mu_\alpha(a)+\pi_3\right)I_{3,\alpha}^S(a,i)$$

Vaccinated population:

$$\frac{dS_\alpha^V(a,i)}{dt}=\eta(a-1)S_\alpha^V(a-1,i)+\gamma_P(a,i)S_\alpha(a,i)-\left(\eta(a)+\mu_\alpha(a)+(1-VE_S)\Lambda_\alpha(a,i)+\varepsilon_P(a,i)\right)S_\alpha^V(a,i)$$

$$\frac{dI_{1,\alpha}^{A,V}(a,i)}{dt}=\left(1-VE_S\right)\left(1-f_S\right)\Lambda_\alpha(a,i)S_\alpha^V(a,i)+\eta(a-1)I_{1,\alpha}^{A,V}(a-1,i)-\left(\eta(a)+\mu_\alpha(a)+\pi_1\right)I_{1,\alpha}^{A,V}(a,i)$$

$$\frac{dI_{2,\alpha}^{A,V}(a,i)}{dt}=\pi_1 I_{1,\alpha}^{A,V}(a,i)+\eta(a-1)I_{2,\alpha}^{A,V}(a-1,i)+\pi_3 I_{3,\alpha}^{A,V}(a,i)-\left(\eta(a)+\mu_\alpha(a)+\pi_2\right)I_{2,\alpha}^{A,V}(a,i)$$

$$\frac{dI_{3,\alpha}^{A,V}(a,i)}{dt}=\pi_2 I_{2,\alpha}^{A,V}(a,i)+\eta(a-1)I_{3,\alpha}^{A,V}(a-1,i)-\left(\eta(a)+\mu_\alpha(a)+\pi_3\right)I_{3,\alpha}^{A,V}(a,i)$$

$$\frac{dI_{1,\alpha}^{S,V}(a,i)}{dt}=(1-VE_S)\,f_S\Lambda_\alpha(a,i)S_\alpha^V(a,i)+\eta(a-1)I_{1,\alpha}^{S,V}(a-1,i)-\left(\eta(a)+\mu_\alpha(a)+\pi_1\right)I_{1,\alpha}^{S,V}(a,i)$$

$$\frac{dI_{2,\alpha}^{S,V}(a,i)}{dt}=\pi_1 I_{1,\alpha}^{S,V}(a,i)+\eta(a-1)I_{2,\alpha}^{S,V}(a-1,i)+\pi_3 I_{3,\alpha}^{S,V}(a,i)-\left(\eta(a)+\mu_\alpha(a)+\pi_2\right)I_{2,\alpha}^{S,V}(a,i)$$

$$\frac{dI_{3,\alpha}^{S,V}(a,i)}{dt}=\pi_2 I_{2,\alpha}^{S,V}(a,i)+\eta(a-1)I_{3,\alpha}^{S,V}(a-1,i)-\left(\eta(a)+\mu_\alpha(a)+\pi_3\right)I_{3,\alpha}^{S,V}(a,i)$$

Figure S2. Schematic diagram describing the structure of the HSV-2 mathematical model incorporating a therapeutic vaccine.

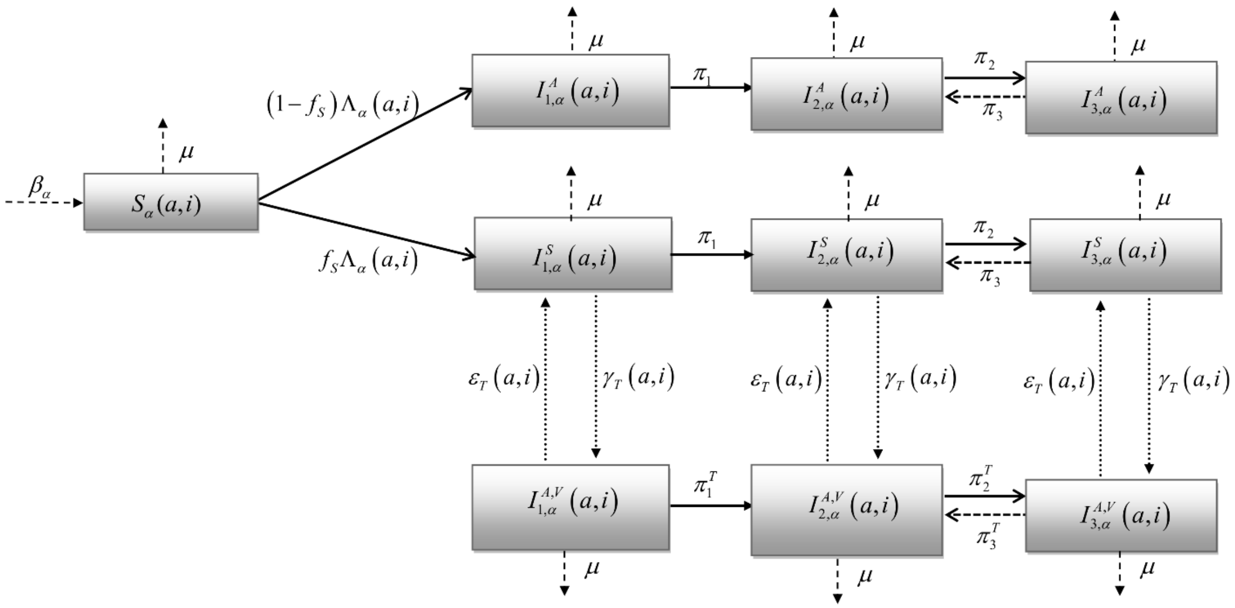

The therapeutic HSV-2 vaccine model was expressed using the following equations:

$$\frac{dS_\alpha(a,i)}{dt}=\beta_\alpha(t,a)N_\alpha(i)+\eta(a-1)S_\alpha(a-1,i)-\left(\eta(a)+\mu_\alpha(a)+\Lambda_\alpha(a,i)\right)S_\alpha(a,i)$$

$$\frac{dI_{1,\alpha}^{A}(a,i)}{dt}=(1-f_S)\Lambda_\alpha(a,i)S_\alpha(a,i)+\eta(a-1)I_{1,\alpha}^{A}(a-1,i)-\left(\eta(a)+\mu_\alpha(a)+\pi_1\right)I_{1,\alpha}^{A}(a,i)$$

$$\frac{dI_{2,\alpha}^{A}(a,i)}{dt}=\pi_1 I_{1,\alpha}^{A}(a,i)+\eta(a-1)I_{2,\alpha}^{A}(a-1,i)+\pi_3 I_{3,\alpha}^{A}(a,i)-\left(\eta(a)+\mu_\alpha(a)+\pi_2\right)I_{2,\alpha}^{A}(a,i)$$

$$\frac{dI_{3,\alpha}^{A}(a,i)}{dt}=\pi_2 I_{2,\alpha}^{A}(a,i)+\eta(a-1)I_{3,\alpha}^{A}(a-1,i)-\left(\eta(a)+\mu_\alpha(a)+\pi_3\right)I_{3,\alpha}^{A}(a,i)$$

$$\frac{dI_{1,\alpha}^{S}(a,i)}{dt}=f_S\Lambda_\alpha(a,i)S_\alpha(a,i)+\eta(a-1)I_{1,\alpha}^{S}(a-1,i)+\varepsilon_T(a,i)I_{1,\alpha}^{S,V}(a,i)-\left(\eta(a)+\mu_\alpha(a)+\pi_1+\gamma_T(a,i)\right)I_{1,\alpha}^{S}(a,i)$$

$$\frac{dI_{2,\alpha}^{S}(a,i)}{dt}=\pi_1 I_{1,\alpha}^{S}(a,i)+\eta(a-1)I_{2,\alpha}^{S}(a-1,i)+\pi_3 I_{3,\alpha}^{S}(a,i)+\varepsilon_T(a,i)I_{2,\alpha}^{S,V}(a,i)-\left(\eta(a)+\mu_\alpha(a)+\pi_2+\gamma_T(a,i)\right)I_{2,\alpha}^{S}(a,i)$$

$$\frac{dI_{3,\alpha}^{S}(a,i)}{dt}=\pi_2 I_{2,\alpha}^{S}(a,i)+\eta(a-1)I_{3,\alpha}^{S}(a-1,i)+\varepsilon_T(a,i)I_{3,\alpha}^{S,V}(a,i)-\left(\eta(a)+\mu_\alpha(a)+\pi_3+\gamma_T(a,i)\right)I_{3,\alpha}^{S}(a,i)$$

Vaccinated population:

$$\frac{dI_{1,\alpha}^{S,V}(a,i)}{dt}=f_S\Lambda_\alpha(a,i)S_\alpha^{V}(a,i)+\eta(a-1)I_{1,\alpha}^{S,V}(a-1,i)+\gamma_T(a,i)I_{1,\alpha}^{S}(a,i)-\left(\eta(a)+\mu_\alpha(a)+\pi_1^{T}+\varepsilon_T(a,i)\right)I_{1,\alpha}^{S,V}(a,i)$$

$$\frac{dI_{2,\alpha}^{S,V}(a,i)}{dt}=\pi_1^{T} I_{1,\alpha}^{S,V}(a,i)+\eta(a-1)I_{2,\alpha}^{S,V}(a-1,i)+\pi_3^{T} I_{3,\alpha}^{S,V}(a,i)+\gamma_T(a,i)I_{2,\alpha}^{S}(a,i)-\left(\eta(a)+\mu_\alpha(a)+\pi_2^{T}+\varepsilon_T(a,i)\right)I_{2,\alpha}^{S,V}(a,i)$$

$$\frac{dI_{3,\alpha}^{S,V}(a,i)}{dt}=\pi_2^{T} I_{2,\alpha}^{S,V}(a,i)+\eta(a-1)I_{3,\alpha}^{S,V}(a-1,i)+\gamma_T(a,i)I_{3,\alpha}^{S}(a,i)-\left(\eta(a)+\mu_\alpha(a)+\pi_3^{T}+\varepsilon_T(a,i)\right)I_{3,\alpha}^{S,V}(a,i)$$

In order to accommodate the heterogeneity in the risk of exposure to HSV-2 infection, we stratified the population by sex ($\alpha =$ male (m), female (f)), as well as 20 age groups ($n_a = 20$; $a = 1, 2, ..., 20$; each representing a five-year age band: $0-4, 5-9, ..., 95-99$), and five sexual risk groups ($n_r = 5$; $i = 1, 2, ..., 5$; indicating low to higher risk of HSV-2 exposure).

In each sex, age, and risk group, the population was further stratified into epidemiological categories that comprise the HSV-2 susceptible populations $S_{\alpha}\left(a,i\right)$, asymptomatic infected populations $I_{\kappa,\alpha}^{A}\left(a,i\right)$, and symptomatic infected populations $I_{\kappa,\alpha}^{S}\left(a,i\right)$. The index κ defines the stage of HSV-2 infection: primary infection ($\kappa=1$), latent infection ($\kappa=2$), and infection reactivation ($\kappa=3$). During primary infection and infection reactivation, individuals can shed the virus and were assumed infectious. However, individuals in the latent infection stage were assumed not infectious. The study investigated the impact of: 1) a prophylactic partially-efficacious vaccine—only susceptible individuals are vaccinated (and progress through the stages of asymptomatic or symptomatic infections if infected despite being vaccinated; Figure S1), and 2) a therapeutic vaccine—only infected symptomatic individuals are vaccinated and the vaccine clears their symptoms and makes their infection effectively asymptomatic (and progress through the stages of asymptomatic infection; Figure S2).

Definitions of all symbols in the equations can be found in Table S1.

Table S1. Definitions of symbols in the equations of the mathematical models.

Symbol	Definition
$N_{\alpha}(i)$	Population size
$S_{\alpha}(a,i)$	HSV-2 susceptible population
$I_{1,\alpha}^{A}\left(a,i\right)$	Asymptomatically infected and infectious population (HSV-2 primary infection)
$I_{2,\alpha}^{A}\left(a,i\right)$	Asymptomatically and latently infected (non-shedding) population
$I_{3,\alpha}^{A}\left(a,i\right)$	Asymptomatically infected and infectious population (HSV-2 infection reactivation)
$I_{1,\alpha}^{S}\left(a,i\right)$	Symptomatically infected and infectious population (HSV-2 primary infection)
$I_{2,\alpha}^{S}\left(a,i\right)$	Symptomatically and latently infected (non-shedding) population
$I_{3,\alpha}^{S}\left(a,i\right)$	Symptomatically infected and infectious population (HSV-2 infection reactivation)
$S_{\alpha}^{V}(a,i)$	Vaccinated HSV-2 susceptible population through the prophylactic vaccine
$I_{1,\alpha}^{A,V}\left(a,i\right)$	Vaccinated asymptomatically infected and infectious population (HSV-2 primary infection)
$I_{2,\alpha}^{A,V}\left(a,i\right)$	Vaccinated asymptomatically and latently infected (non-shedding) population
$I_{3,\alpha}^{A,V}\left(a,i\right)$	Vaccinated asymptomatically infected and infectious population (HSV-2 infection reactivation)
$I_{1,\alpha}^{S,V}\left(a,i\right)$	Vaccinated symptomatically infected and infectious population (HSV-2 primary infection)

$I_{2,\alpha}^{S,V}(a,i)$	Vaccinated symptomatically and latently-infected (non-shedding) population
$I_{3,\alpha}^{S,V}(a,i)$	Vaccinated symptomatically infected and infectious population (HSV-2 infection reactivation)
$\beta_\alpha(t,a)$	Population growth rate, where $\beta_\alpha(t,1)=\tilde{\beta}_\alpha$ and $\beta_\alpha(t,a>1)=0$
$\mu_\alpha(a)$	Natural mortality rate
$\eta(a)$	Transition rate from one age group to the next age group, where $\eta(a<0)=0$ and $\eta(a>0)=\tilde{\eta}(a)$
f_S	Proportion of infections that are symptomatic
$\Lambda_\alpha(a,i)$	HSV-2 force of infection experienced by each susceptible $S_\alpha(a,i)$ and $S_\alpha^V(a,i)$ population
π_1	Rate of progression from the primary to the latent stage (no or prophylactic vaccination)
π_2	Rate of progression from the latent to the reactivation stage (no or prophylactic vaccination)
π_3	Rate of progression from the reactivation to the latent stage (no or prophylactic vaccination)
π_1^T	Rate of progression from the primary to the latent stage (therapeutic vaccination)
π_2^T	Rate of progression from the latent to the reactivation stage (therapeutic vaccination)
π_3^T	Rate of progression from the latent to the reactivation stage (therapeutic vaccination)
$\gamma_P(a,i)$	Rate at which susceptible individuals are vaccinated with the prophylactic vaccine
$\varepsilon_P(a,i)$	Rate at which vaccination immunity wanes for individuals who were vaccinated with the prophylactic vaccine
$\gamma_T(a,i)$	Rate at which symptomatically infected individuals are vaccinated with the therapeutic vaccine
$\varepsilon_T(a,i)$	Rate at which vaccination immunity wanes for symptomatically infected individuals who were vaccinated with the therapeutic vaccine
VE_S	Proportional reduction in the susceptibility to infection upon vaccination with the prophylactic vaccine, relative to those unvaccinated
VE_P	Proportional reduction in shedding frequency upon vaccination with the therapeutic vaccine, relative to those unvaccinated

The transition of the population from one age group to the next age group was represented by the transition rate η. The progression rates between the HSV-2 infection stages were described by π_1, π_2 and π_3 in case of no-vaccination or in case of the introduction of a prophylactic vaccine, and by π_1^T, π_2^T and π_3^T in case of the introduction of a therapeutic vaccine.

The population growth rate (β_α) was described using

$$\beta_\alpha(t)=b_{0,\alpha}\, e^{-\left(\frac{t-b_{1,\alpha}}{b_{2,\alpha}}\right)^2}$$

Meanwhile, the natural mortality rate (μ_α) was described using

$$\mu_\alpha(t,a) = \frac{c_{0,\alpha}\; e^{-\left(\frac{t-c_{1,\alpha}}{c_{2,\alpha}}\right)^2}}{\left[1+e^{-\left(c_{3,\alpha}\left(a-c_{4,\alpha}\right)\right)}\right]}$$

These functions were chosen as they produced a robust fit of the population growth and age structure in the United States (US) [2]. The demographic parameters $b_{0,\alpha}$, $b_{1,\alpha}$, $b_{2,\alpha}$, $c_{0,\alpha}$, $c_{1,\alpha}$, $c_{2,\alpha}$, $c_{3,\alpha}$, and $c_{4,\alpha}$ were determined through model fitting [1].

The structure, subfunctions, and details of the HSV-2 force of infection $\Lambda_\alpha(a,i)$ experienced by each $S_\alpha(a,i)$ and $S_\alpha^V(a,i)$ susceptible population can be found in Reference [1].

2. Parameter values

Model parameters were based on current empirical data for HSV-2 natural history and transmission, and for population sexual risk behavior. Table S2 provides a listing of these parameters. Further details on the model parametrization can be found in Reference [1].

Table S2. Model assumptions in terms of parameter values.

Parameter	Symbol	Value	Justification	Sources
Transmission probability per coital act *in primary* $(\kappa = 1)$ and *reactivation* $(\kappa = 3)$ stages		0.01	Combination of empirical data and quantitative estimates	[1,3]
HSV-2 shedding frequency	ξ	18.3% of the time	Direct measurement from a prospective cohort study	[4]
Frequency of HSV-2 reactivations	χ	15 per year	Direct measurement from a prospective cohort study	[4]
Duration of primary infection	$\upsilon_1 = \frac{1}{\pi_1}$	20.0 days	Representative assumption informed by data	[5]
Duration of latency between two reactivations	$\upsilon_2 = \frac{1}{\pi_2}$	19.8 days	Derived: $\upsilon_2 = \left(\frac{1-\xi}{\chi}\right) \times 365$	[3,6]
Duration of reactivation within the cycle	$\upsilon_3 = \frac{1}{\pi_3}$	4.4 days	Derived: $\upsilon_3 = \left(\frac{\xi}{\chi}\right) \times 365$	[3,6]
Degree of assortativeness for age group mixing		0.7	Informed by infectious disease modeling works	[1,7,8] and representative value
Degree of assortativeness for risk group mixing		0.3	Informed by infectious disease modeling works	[1,7,8] and representative value
Proportion of infections that are symptomatic	f_S	0.25	Informed by totality of evidence from different studies	[4,5,9-12]
Proportional reduction in the susceptibility to infection upon vaccination, relative to those unvaccinated	VE_S	0 – 100%	Explored values	Representative values
Proportional reduction in the frequency of shedding upon vaccination, relative to those unvaccinated	VE_P	0-100%	Explored values	Representative values
Duration of primary infection for those vaccinated	$\upsilon_{1,V} = \frac{1}{\pi_{1,V}}$	20.0 days	Assumption informed by data and assuming vaccination does not affect primary infection duration	[5]

Duration of latency between two reactivations for those vaccinated	$\upsilon_{2,V} = \frac{1}{\pi_{2,V}}$	19.9-24.3 days	Derived: $\upsilon_2 = \left(\frac{1-(1-VE_P)\xi}{\chi}\right)\times 365$	[3,6]
Duration of reactivation within the cycle for those vaccinated	$\upsilon_{3,V} = \frac{1}{\pi_{3,V}}$	0-4.4 days	Derived: $\upsilon_3 = \left(\frac{(1-VE_P)\xi}{\chi}\right)\times 365$	[3,6]
Duration of the prophylactic vaccine protection	$\frac{1}{\varepsilon_P}$	5-30 years	Explored values	Representative values
Duration of the therapeutic vaccine protection	$\frac{1}{\varepsilon_T}$	5-30 years	Explored values	Representative values

Figure S3. Impact of prophylactic HSV-2 vaccination administered to susceptible women aged 15-49 years (single-sex vaccination) on HSV-2 infection measures in the population aged ≥15 years. Impact of the prophylactic vaccine on (**A**) annual number of new HSV-2 infections, (**B**) annual number of HSV-2 infections averted, (**C**) HSV-2 incidence rate, and (**D**) HSV-2 seroprevalence, among those aged ≥ 15 years. The prophylactic vaccine is introduced in 2020, with its coverage scaled up to 80% by 2030, and maintained at this level thereafter. Duration of vaccine-induced protection is 20 years and VE_S is 50%.

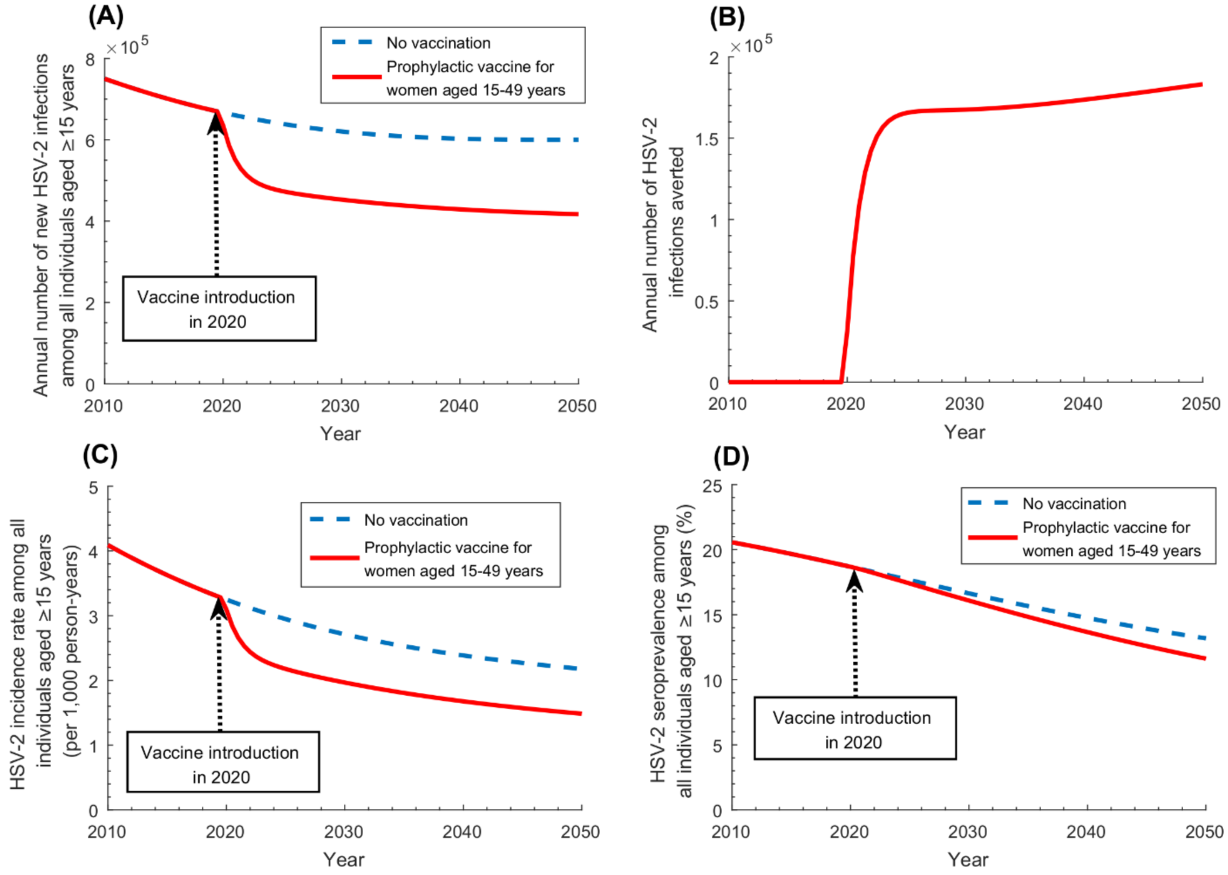

Figure S4. Impact of prophylactic HSV-2 vaccination administered to infants (infants' vaccination) on HSV-2 infection measures in the population aged ≥15 years. Impact of the prophylactic vaccine on (**A**) annual number of new HSV-2 infections, (**B**) annual number of HSV-2 infections averted, (**C**) HSV-2 incidence rate, and (**D**) HSV-2 seroprevalence, among those aged ≥ 15 years. The prophylactic vaccine is introduced in 2020, with its coverage scaled up to 80% by 2030, and maintained at this level thereafter. Duration of vaccine-induced protection is 30 years and *VEs* is 50%.

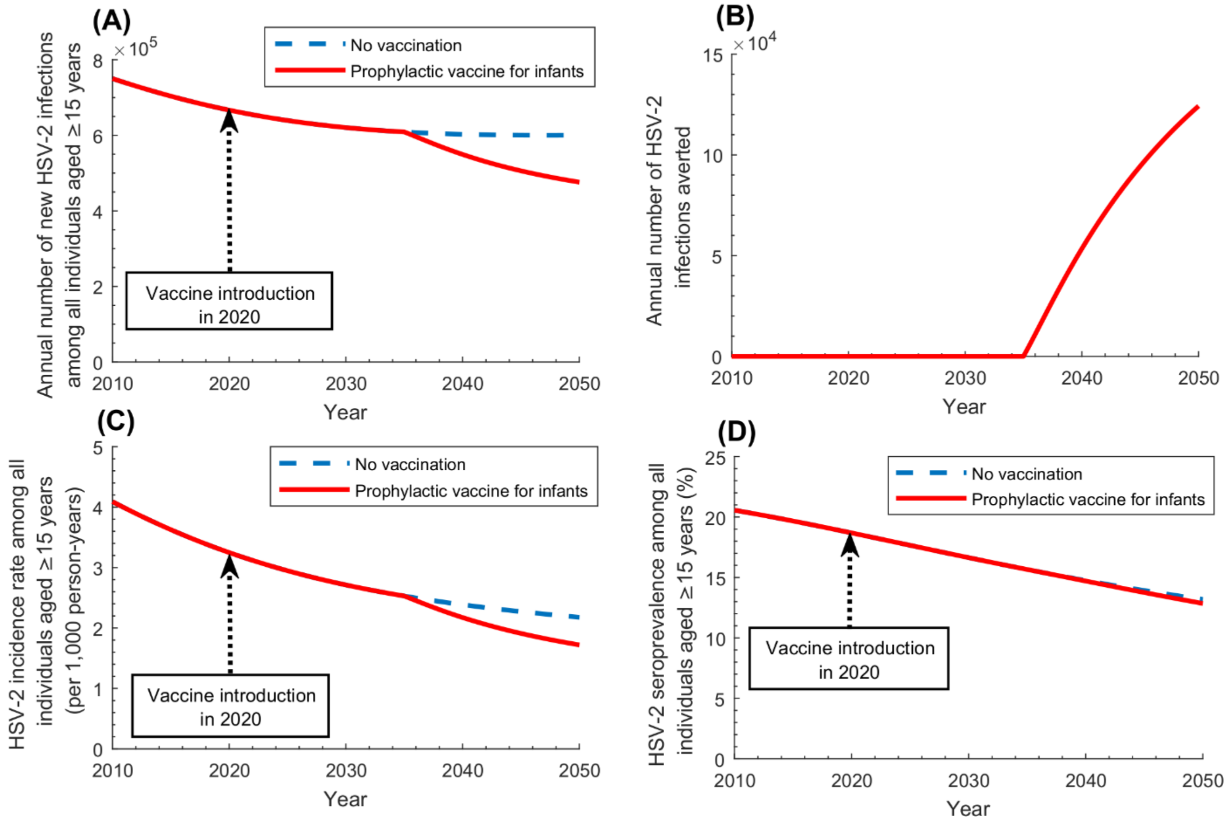

Figure S5. Sensitivity analysis assessing the effectiveness of prophylactic and therapeutic vaccination to a range of vaccine efficacies. Number of vaccinations needed to avert one infection by 2050 at various levels of (**A**) prophylactic vaccine efficacy VE_S, and (**B**) therapeutic vaccine efficacy VE_P. Vaccines are introduced in 2020, with their coverage scaled-up to 80% at 2030, and maintained at this level thereafter. Duration of vaccine-induced protection is 20 years for the prophylactic vaccine and 10 years for the therapeutic vaccine.

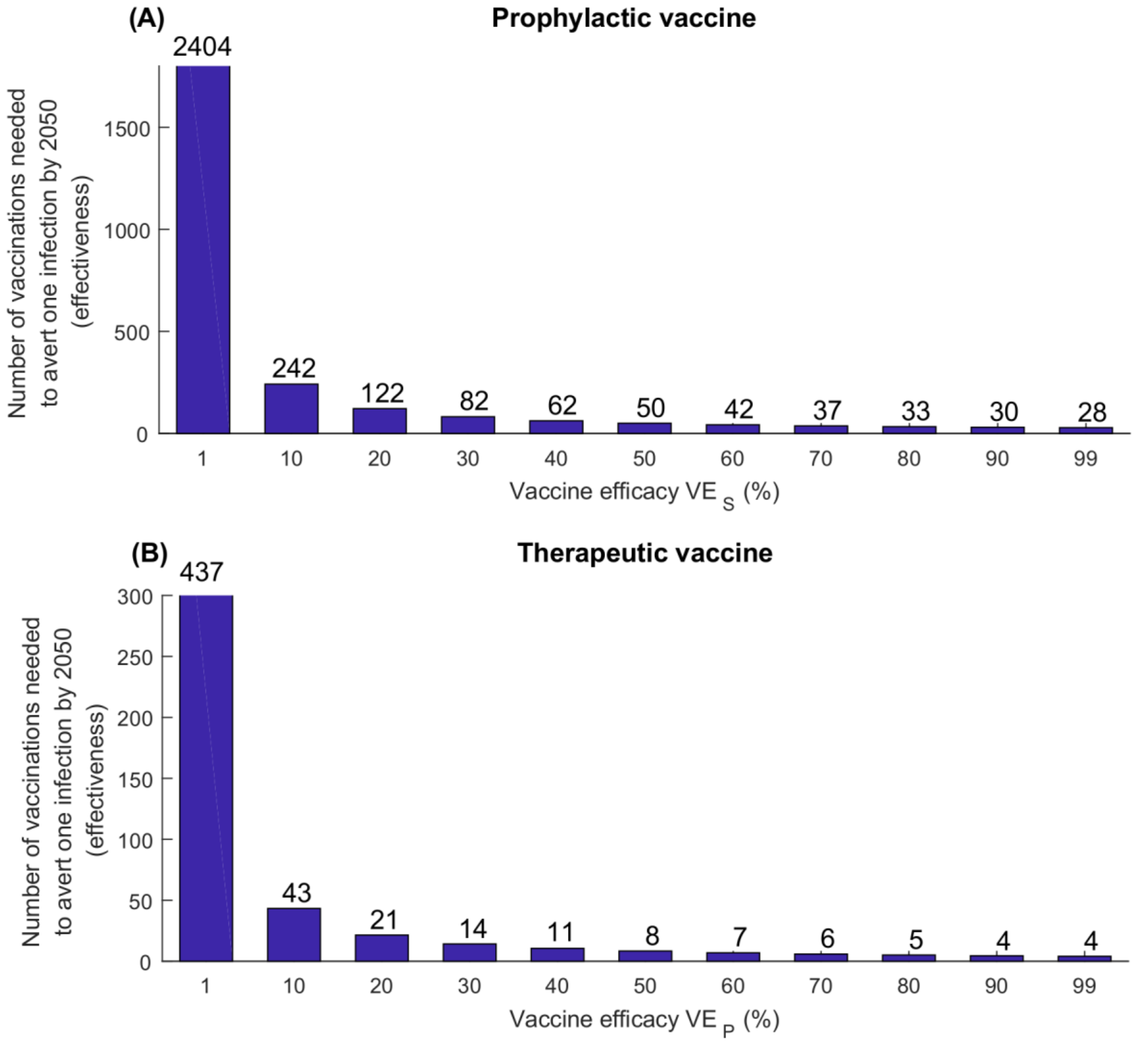

Figure S6. Sensitivity analysis assessing the effectiveness of prophylactic and therapeutic vaccination to a range of vaccine-induced protection durations. Number of vaccinations needed to avert one infection by 2050 at various levels of (**A**) prophylactic vaccine protection duration, and (**B**) therapeutic vaccine protection duration. Vaccines are introduced in 2020, with their coverage scaled-up to 80% at 2030, and maintained at this level thereafter. VE_S is 50% and VE_P is 50%.

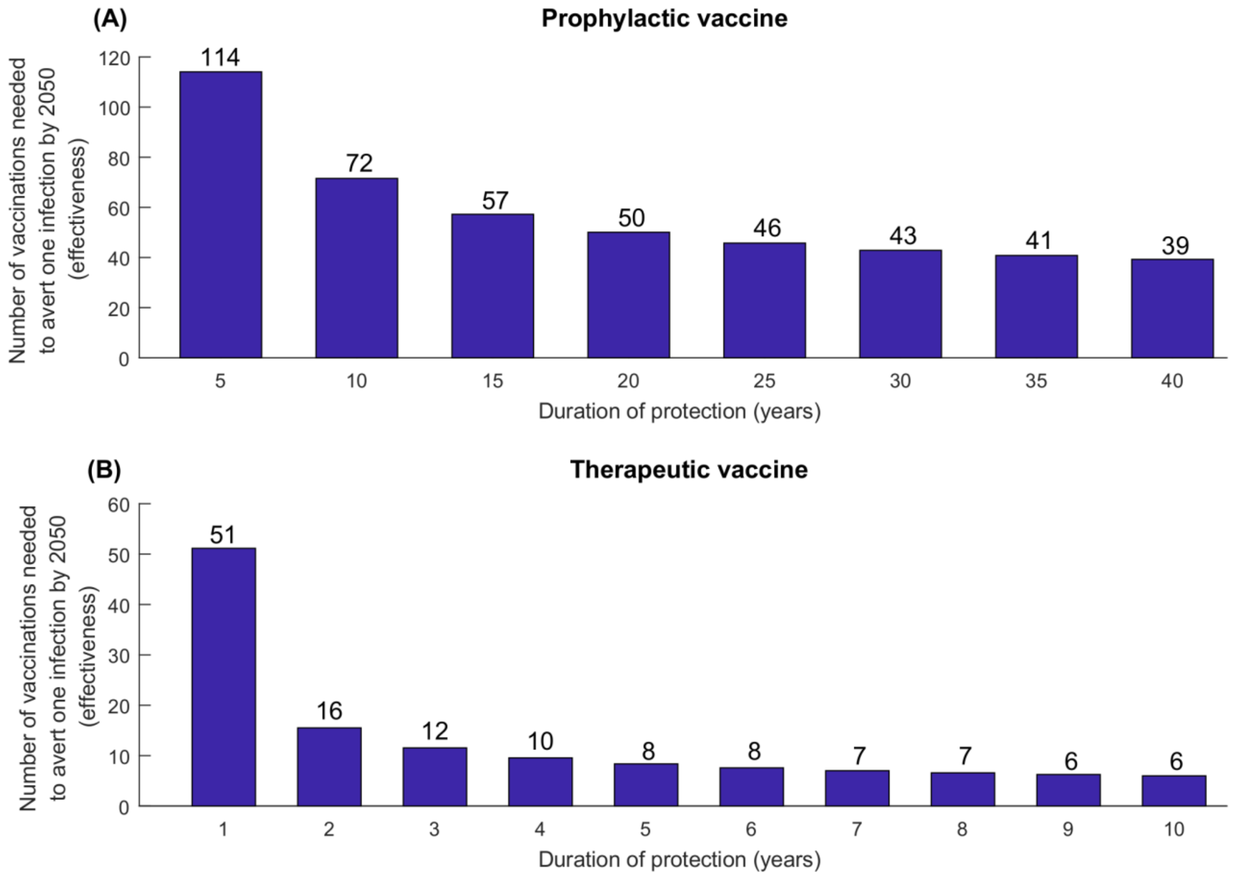

Figure S7. Vaccine coverage of (**A**) the catch-up prophylactic vaccination scenario, and (**B**) the therapeutic vaccination scenario.

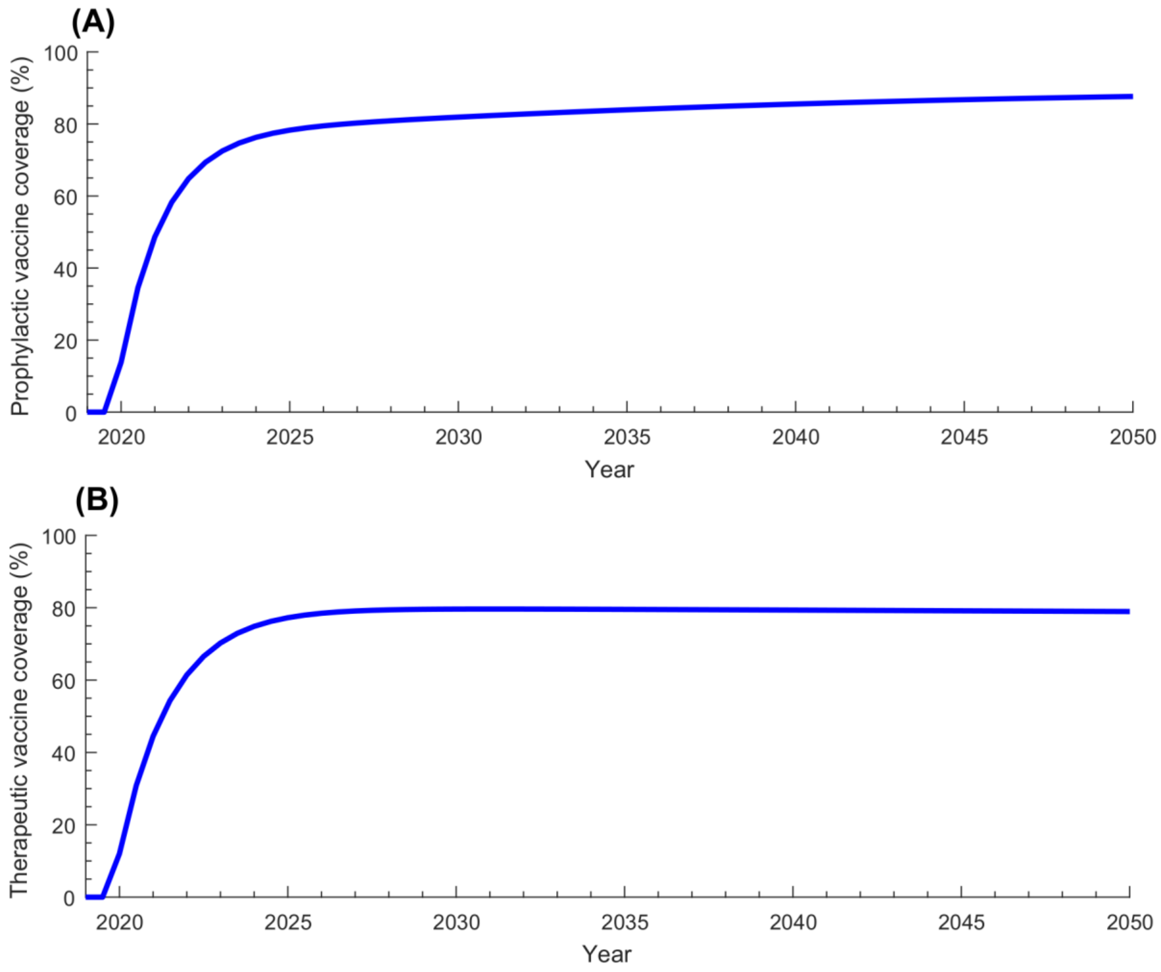

Figure S8. Impact of prophylactic HSV-2 vaccination administered to uninfected adults aged 15-49 years (catch-up vaccination) on the reduction in the annual number of new HSV-2 infections assuming different levels of vaccine coverage. Duration of vaccine-induced protection is 20 years and VE_S is 50%.

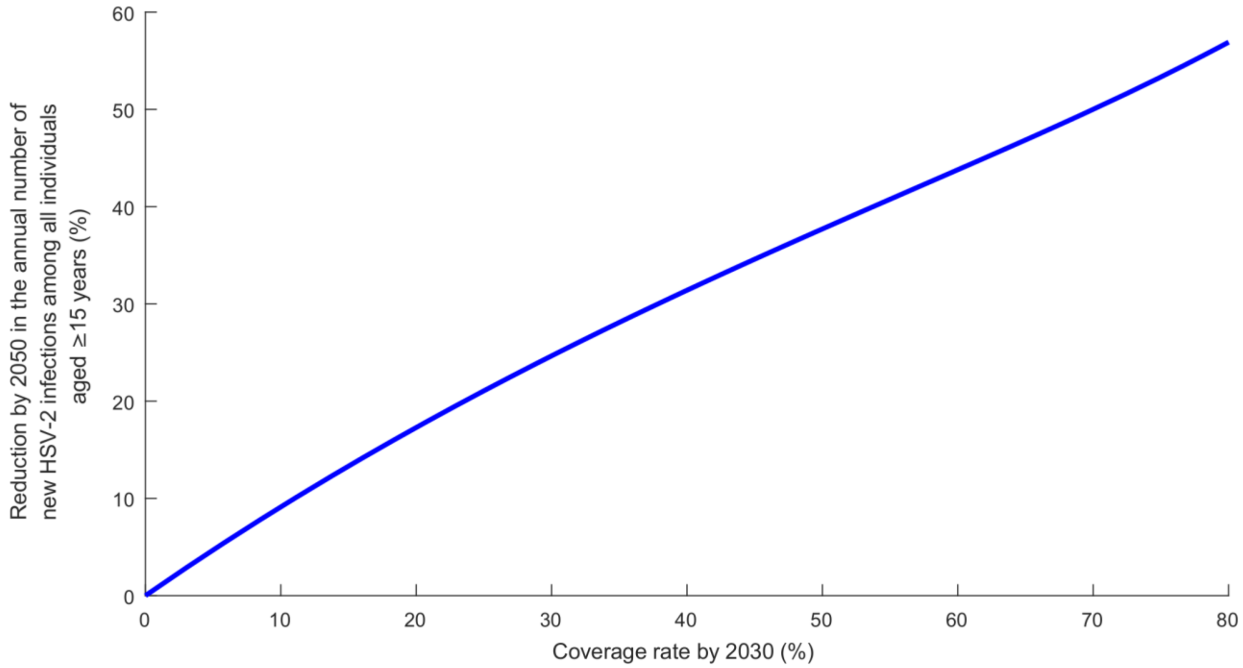

Figure S9. Uncertainty analysis. Model predictions for the mean number of vaccinations needed to avert one infection, and associated 95% uncertainty interval (UI), using (**A**) a prophylactic vaccine, and (**B**) a therapeutic vaccine. Vaccines are introduced in 2020, with their coverage scaled-up to 80% at 2030, and maintained at this level thereafter. VE_S is 50% and VE_P is 50%. Duration of vaccine-induced protection is 20 years for the prophylactic vaccine and 10 years for the therapeutic vaccine.

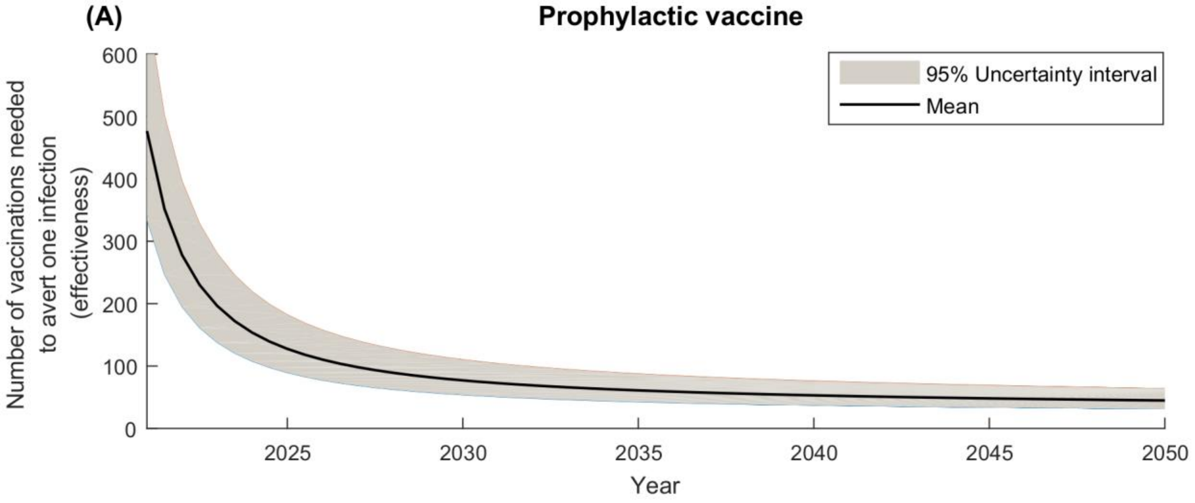

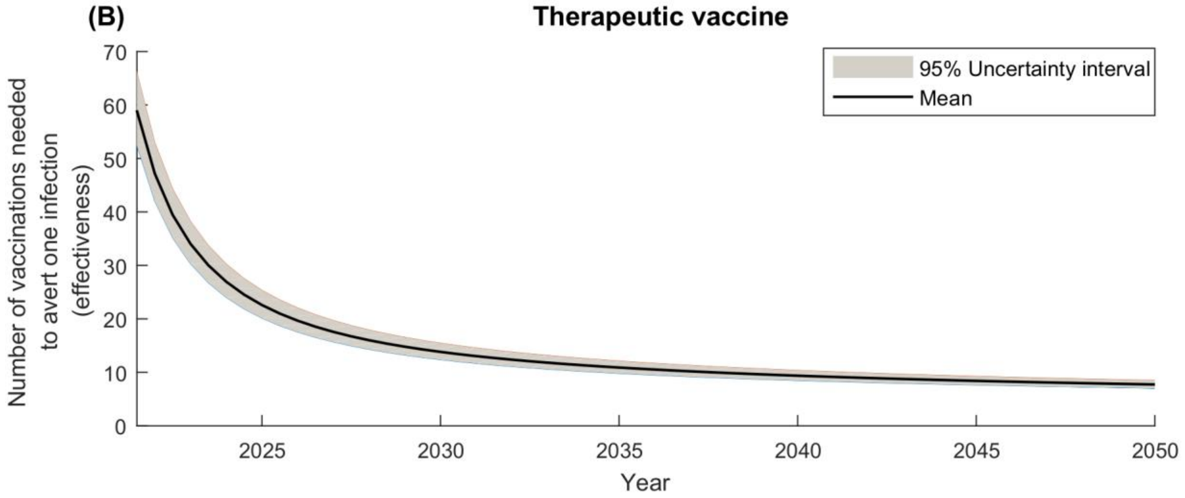

References

1. Ayoub, H.H.; Amara, I.; Awad, S.F.; Chemaitelly, H.; Abu-Raddad, L.J. Analytic characterization of the herpes simplex virus type 2 epidemic in the United States, 1950-2050. Under Review. **under preparation**.
2. United Nations Department of Economic and Social Affairs. World Population Prospects, the 2015 Revision. 2015.
3. Abu-Raddad, L.J.; Magaret, A.S.; Celum, C.; Wald, A.; Longini, I.M., Jr.; Self, S.G.; Corey, L. Genital herpes has played a more important role than any other sexually transmitted infection in driving HIV prevalence in Africa. *PLoS One* **2008**, *3*, e2230, doi:10.1371/journal.pone.0002230.
4. Tronstein, E.; Johnston, C.; Huang, M.L.; Selke, S.; Magaret, A.; Warren, T.; Corey, L.; Wald, A. Genital shedding of herpes simplex virus among symptomatic and asymptomatic persons with HSV-2 infection. *JAMA* **2011**, *305*, 1441-1449, doi:10.1001/jama.2011.420.
5. Benedetti, J.; Corey, L.; Ashley, R. Recurrence rates in genital herpes after symptomatic first-episode infection. *Ann Intern Med* **1994**, *121*, 847-854.
6. Alsallaq, R.A.; Schiffer, J.T.; Longini, I.M., Jr.; Wald, A.; Corey, L.; Abu-Raddad, L.J. Population level impact of an imperfect prophylactic vaccine for herpes simplex virus-2. *Sex Transm Dis* **2010**, *37*, 290-297, doi:10.1097/OLQ.0b013e3181d3d023.
7. Awad, S.F.; Sgaier, S.K.; Tambatamba, B.C.; Mohamoud, Y.A.; Lau, F.K.; Reed, J.B.; Njeuhmeli, E.; Abu-Raddad, L.J. Investigating Voluntary Medical Male Circumcision Program Efficiency Gains through Subpopulation Prioritization: Insights from Application to Zambia. *PLoS One* **2015**, *10*, e0145729, doi:10.1371/journal.pone.0145729.
8. Ayoub, H.H.; Chemaitelly, H.; Abu-Raddad, L.J. Characterizing the transitioning epidemiology of herpes simplex virus type 1 in the USA: model-based predictions. *BMC medicine* **2019**, *17*, 57.
9. Fleming, D.T.; McQuillan, G.M.; Johnson, R.E.; Nahmias, A.J.; Aral, S.O.; Lee, F.K.; St Louis, M.E. Herpes simplex virus type 2 in the United States, 1976 to 1994. *N Engl J Med* **1997**, *337*, 1105-1111, doi:10.1056/NEJM199710163371601.
10. Langenberg, A.G.; Corey, L.; Ashley, R.L.; Leong, W.P.; Straus, S.E. A prospective study of new infections with herpes simplex virus type 1 and type 2. Chiron HSV Vaccine Study Group. *N Engl J Med* **1999**, *341*, 1432-1438, doi:10.1056/NEJM199911043411904.
11. Wald, A.; Zeh, J.; Selke, S.; Ashley, R.L.; Corey, L. Virologic characteristics of subclinical and symptomatic genital herpes infections. *N Engl J Med* **1995**, *333*, 770-775, doi:10.1056/NEJM199509213331205.
12. Langenberg, A.; Benedetti, J.; Jenkins, J.; Ashley, R.; Winter, C.; Corey, L. Development of clinically recognizable genital lesions among women previously identified as having "asymptomatic" herpes simplex virus type 2 infection. *Ann Intern Med* **1989**, *110*, 882-887, doi:10.7326/0003-4819-110-11-882.
